# Genome agnostic, multi-level non-oncogene addiction-based systems pharmacology for rescuing metastatic relapsed/refractory neoplasias

**DOI:** 10.3389/fphar.2026.1629187

**Published:** 2026-02-24

**Authors:** Dennis Christoph Harrer, Florian Lüke, Tobias Pukrop, Lina Ghibelli, Albrecht Reichle, Daniel Heudobler

**Affiliations:** 1 Department of Internal Medicine III, Hematology and Oncology, University Hospital Regensburg, Regensburg, Germany; 2 Division of Personalized Tumor Therapy, Fraunhofer Institute for Toxicology and Experimental Medicine, Regensburg, Germany; 3 Bavarian Cancer Research Center (BZKF), University Hospital Regensburg, Regensburg, Germany; 4 Department of Biology, University of Rome “Tor Vergata”, Rome, Italy

**Keywords:** acute myelocytic leukemia, all-trans retinoic acid, anakoinosis, angiosarcoma, azacitidine, cancer hallmarks, dexamethasone, Hodgkin’s lymphoma

## Abstract

Rescue therapies for relapsed/refractory (r/r) metastatic neoplasias present significant unmet needs. Tumor tissue editing regimen for 13 r/r tumor types, carcinomas, sarcomas and hematologic neoplasias, included in 15 phase I/II trials, nuclear/cytokine receptor agonists, pioglitazone, plus/minus dexamethasone or all-trans retinoic acid or interferon-α to counterbalance tumor tissue homeostasis and reprogramming of cancer hallmarks, stress response inhibitors, COX-2 inhibitor, everolimus, lenalidomide, or clarithromycin, and a stress response inducer, low-dose metronomic chemotherapy with treosulfan, trofosfamide, capecitabine, or azacitidine. CR in three, cCR in another five r/r neoplasias, as the best response occurred after transcriptional reprogramming of cancer hallmarks, inflammation control or differentiation induction. Receptor agonist combinations for cCR induction can be identical among quite different tumor types and diversified within the same tumor histology. Data reveal ubiquitous, differential transcriptional access to non-oncogene addiction (NOA) networks that cope with cancer hallmarks/stress responses and three levels of therapeutic NOA targeting. (1) Agonists of nuclear/cytokine receptor NOAs critically target tumor identity and viability, while (2) transcriptional reprogramming of NOA networks that contribute to tumor tissue addiction, thereby genome-agnostically counteracting oncogene addictions. (3) Targeting edited NOAs may improve long-term outcome with CR/cCR (everolimus, IMiD). Transcriptionally accessible NOA targets offer high specificity, modest toxicity profile, low cost of therapy and outpatient treatment, independent of comorbidities. Adaptive targeting of the transcriptomic landscapes of tumor cell compartments breaks tumor tissue addiction and overcomes M-CRAC, post-therapy metastasis, cancer cell recolonization, acquired resistance and genetic heterogeneity. Thus, editing approaches provide a template for controlling metastatic r/r tumors. In the future, diagnostics of NOA networks and transcription factors involved in tumor tissue addiction may be as valuable for therapy selection as histological/molecular genetic tumor typing for the establishment of personalized hematology/oncology.

## Introduction

The treatment of metastatic recurrent/refractory (r/r) neoplasias is a challenge for any systemic therapy.

Common oncogenic drivers in histologically diverse tumor types allow the use of tumor type-independent drugs targeting oncogene addiction (Thein et al., 2024; Yılmaz et al., 2024; Mechahougui et al., 2024). The lack of single oncogenic drivers in most neoplasias and the heterogeneous genetic aberrations at metastatic sites make the selection of therapeutic targets challenging when cure, rather than palliation, is the therapeutic goal (Vogelstein et al., 2013). In most cases, the choice of treatment is empirical and based on clinically selected sequences of trial designs (Sibai et al., 2022; Hendriks et al., 2023). Oncogenic events and their translation into tumor phenotypes are poorly understood by mutations alone, based on novel crossovers with the germline genome, protein-protein rewiring, re-specified effects of signaling pathways, and extensive remodeling of the tumor microenvironment (Sibai et al., 2022; Swanton et al., 2024).

As therapeutic targets, non-oncogene addictions (NOAs) are emerging as therapeutically accessible molecules to break tumor tissue addiction. The concept of tumor tissue addiction refers to the phenomenon that cancer hallmarks and stress responses configure tumor disease characteristics that provide key pharmacological targets (Baron et al., 2020; Swanton et al., 2024; Harrer et al., 2023b).

We view tumor-specific, functionally integrated cancer hallmarks and stress responses as multilevel addictions, initially initiated by oncogenic events, but ultimately dependent on transcriptionally tightly regulated wild-type genes that support pathways, hubs, and rate-limiting NOAs, but also NOA circuitries (Table 1), to maintain tumor viability and tumor stress relief (Bradner et al., 2017; Califano, 2011).

**TABLE 1:** Boxed definitions.

Term	Definitions
Tumor tissue addictions	• Tumor-specific, functionally integrated cancer hallmarks and stress responses are considered as multilevel addictions, initially initiated by oncogenic events, but ultimately dependent on transcriptionally tightly regulated wild-type genes that support pathways, hubs, and rate-limiting NOAs, but also NOA circuitries
Tumor tissue editing therapy	• NOA-based reprogramming of tumor promoting cancer hallmarks into “normalized” tumor growth controlling hallmarks and attenuation/resolution of M-CRAC (Table 3)
​	• Examples: Low dose metronomic chemotherapy or azacitidine or targeted therapies (e.g., MEK inhibitors, immunotherapies) combined with agonists of receptor-triggered transcription factors
​	• Systems pharmacological approach targeting different organizational levels of NOAs covering cancer hallmarks, stress responses and M-CRAC (Figure 2)
​	• Specific receptor agonist combinations for induction of continuous CR can be identical among quite different tumor types and diversified within the same tumor histology
​	• Biological and clinical readouts indicate the reprogramming of tumor-promoting cancer hallmarks by tumor tissue editing therapies
Non-oncogene addiction (NOA) targets	• Oncogenic events induce important communicatively linked NOA targets distributed across tumor tissues
​	• Nuclear and cytokine receptors emerge as key pharmacological targets for reprogramming NOA-driven tissue functions, e.g., tumor promoting hallmarks (Table 2)
Editing of NOA targets	• Clinical response patterns reveal that single NOA targets can be therapeutically edited as rate-limiting targets (currently not studied by direct molecular profiling)
Non-oncogene addiction (NOA) circuitries	• Clinical read-outs indicate that NOAs are modularly organized in NOA-centered communicative systems, so called NOA circuitries, accessible by tumor tissue editing
​	• NOA circuitries are clinically inferred functional modules, not yet quantitatively defined entities
​	• Clinical classification of NOA circuitries: According to the combination of receptor agonists reprogramming cancer hallmarks, or to the type of reprogrammed cancer hallmarks
​	• Accessibility of NOA circuitries is broad but not uniform, and likely context- and circuitry-dependent; shared therapeutic accessibility among histologically different neoplasias
​	• The tool of modularly organized NOA-circuitries is heterogeneous but might be dramatically smaller and more structured than the highly heterogeneous tool of non-synonymous mutations when considering targets for resolving M-CRAC
NOA circuitries/cancer hallmarks	• E.g., differentially operated tools promoting tumor-associated inflammation may share identical therapeutic accessibility via NOA circuitries by tumor tissue editing
Anakoinosis	• Anakoinosis means communicative reprogramming of cancer hallmarks (differentiation induction, inflammation control, enhancement of immunological surveillance, reprogramming of tumor metabolism, and tumor viability)
​	• Anakoinosis directly counteracts the reprogramming capabilities of oncogenes by selectively editing communicatively cross-linked NOA circuitries in tumor tissues
Tumor-specific clusters of tumor tissue addictions	• Tumor-specific clustering response behavior following tumor tissue editing beyond reprogramming of tumor promoting cancer hallmarks (Table 4)
Modular NOA-directed therapy approach	• Modular NOA-directed therapy is characterized by the extent to which NOA circuitries that maintain hallmarks of cancer, stress responses and M-CRAC can be separated and recombined therapeutically by tumor tissue editing approaches for finally disrupting tumor resilience and viability
​	• Pioglitazone, a peroxisome proliferator-activated receptor α/γ agonist, is a prime example of a drug that acts in a modular way, depending on its combination partners
Genome-agnostic	• Induction of complete remission in (molecular-) genetically heterogeneous non-promyelocytic acute myelocytic leukemia (non-PML AML) with an unique AML editing technique
​	• Overcoming intratumor genetic heterogeneity: Resolving mixed responses to previous therapies by editing tumour tissue to induce CR or cCR
​	• Induction of objective reponse CR and cCR independent of the median load of known non-synonymous mutations in the respective histologic tumor types being edited
​	• Induction of CR or cCR using identical tumor tissue editing techniques in histologically different tumors
M-CRAC	• Post-therapy metastasis, cancer cell recolonization, acquired resistance and genetic heterogeneity
Molecular mechanisms promoting tumor tissue editing	• Discussed mechanisms of action of tumor tissue editing therapies are clinically inferred or hypothesis-generating but have not yet been mechanistically determined
​	• *In vitro* data on editing non-PML AML confirm induction of differentiation; reconstitution of the immunocompetence of neutrophils harboring genetic abnormalities of the blasts
​	• A proteomic platform demonstrated how AML differentiation can be achieved; tissue mass spectrometry imaging (MSI) would be an approach to studying NOA circuitries

The addictions become so profound and pervasive that characteristic clusters of addictive tumor functions eventually make up the repertoire of tumor cell-tumor stroma-host interactions that lead to acquired clinical problems that emerge as the sum of oncogenic events which promote transcriptional addiction (Swanton et al., 2024; Bradner et al., 2017). The therapeutically critical question is whether tumor tissue addictions provide extensive NOA circuitries as therapeutic targets for the ultimate control of metastatic r/r neoplasia?

NOAs are commonly used as therapeutic targets for systemic tumor therapy (Di Marco et al., 2024). However, pulsed systemic therapies, including those targeting NOAs administered at maximally tolerated doses, pose a challenge to network-based tumor systems in metastatic r/r neoplasias by promoting stress responses (Hjaltelin et al., 2019; Harrer et al., 2023b). Thus, tumor responses to systemic anticancer therapies can in principle be double-edged: they promote tumor shrinkage, but at the same time initiate a NOA-mediated stress response of the tumor system, M-CRAC, i.e., metastasis, cancer cell repopulation, acquired resistance and tumor cell heterogeneity, a type of stress response that occurs after pulsed apoptosis-inducing chemotherapies or NOA-targeted therapies and severely impairs therapeutic outcome (Harrer et al., 2023b; Weiss et al., 2022; Ma et al., 2016; Chan et al., 2016).

As demonstrated in 15 phase I/II clinical trials in metastatic r/r cancers, sarcomas and hematologic neoplasms, generic nuclear and cytokine receptor NOAs provide targeted receptor agonist access to the transcriptomic landscapes of tumor tissues, enabling specific therapeutic targeting of cellular tumor compartments through selective reprogramming and deregulation of cancer hallmarks and tumor stress responses (Bradner et al., 2017; Harrer et al., 2023b). Nuclear and cytokine receptors emerged as key pharmacological targets for NOAs (Harrer et al., 2023b). Combined targeting with receptor agonists can induce CR and cCR in metastatic r/r neoplasms when their combined activity profile is initiated with low-dose metronomic chemotherapy and/or epigenetic modulation (Lüke et al., 2022; Tao and Wu, 2023; Harrer et al., 2023a; Bushweller, 2019).

Network-based NOA-triggered systems pharmacology of tumors provides important pathophysiological insights into how tumor tissue addictions can be abolished by approved but repurposed metronomically applied agonists of receptor-regulated transcription factors, pioglitazone, all-trans retinoic acid, dexamethasone and interferon-α in appropriate recombinations (Lüke et al., 2022; Vidal, 2020; Dicitore et al., 2014; Harrer et al., 2023b).

This review compares systems pharmacological treatment strategies in metastatic r/r neoplasias, i.e., tumor tissue editing techniques: editing approaches provide a novel template for the control of metastatic r/r neoplasias.

### A novel therapeutic target: the addictive nature of tumor tissue

**FIGURE 1:**
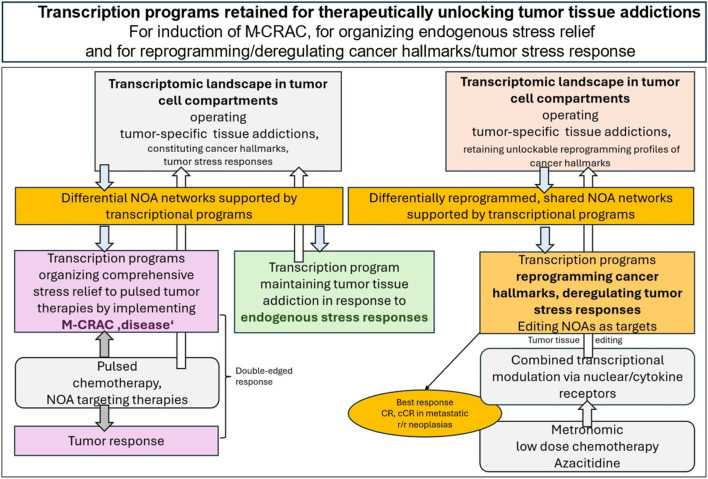
Transcription programs retained for therapeutically unlocking tumor tissue addictions, left: for developing M-CRAC, middle: for tumor stress relief, right: for tumor tissue editing. Tumor plasticity is not only the prerequisite for developing drug resistance but may be therapeutically exploited on basis of well-organized hallmarks of cancer constituted by tumor cells and tumor stroma cells within modular acting circuitries that are accessible to NOA based systems pharmacologic approaches, e.g., tumor tissue editing.

**FIGURE 2:**
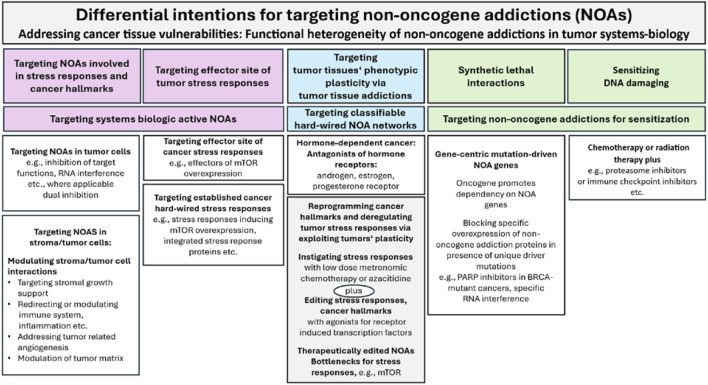
Differential intentions for targeting non-oncogene addictions (NOAs). PARP, Poly-ADP-Ribose-Polymerase; BRCA, Breast CAncer.

Inevitably, therapeutic approaches to control tumor tissue without oncogene addiction, especially in the metastatic r/r stage, favor NOA targets that are genome-agnostic (Hendriks et al., 2023; Heudobler et al., 2024a). Some NOA targets have been shown to be the Achilles’ heel of metastatic neoplasia, as evidenced by pivotal therapeutic outcomes, e.g., with immune checkpoint inhibitors or engineered immunotherapies (Ye et al., 2025) (Table 2; Figure 2). However, therapeutic NOA inhibition can also orchestrate NOA-mediated stress responses in tumors and M-CRAC (Figure 1) (Hjaltelin et al., 2019). M-CRAC develops as an endogenous response to pulsed systemic tumor therapy triggered by the interaction of cancer cells with the adjacent tumor microenvironment (Barkley et al., 2022; Corsi et al., 2022). In addition, unintended off-target effects may limit the use of NOA-targeted therapies (Carneiro and El-Deiry, 2020).

**TABLE 2:** Tumor tissue editing.

Tumor tissue editing
Addictions provide targets for resolving M-CRAC in metastatic tumor disease
Targeting non-oncogene addiction (NOA) regulatory networks that maintain tumor tissue addiction
Networked NOAs can be accessed for therapeutic purposes via
• Editing the transcriptional landscape of tumor cell compartments
• The combined targeting of tumor tissue addictions
• For deregulation of tumor stress responses
• For reprogramming cancer hallmarks and
• Resolving M-CRAC
• Transcriptional modulators with concerted activity profiles
Agonists of nuclear/cytokine receptors
• Dual transcriptional modulation
• Peroxisome proliferator-activated receptorα/γ (PPARα/γ)
• Triple transcriptional modulation
• PPARα/γ/RAR/RXR (retinoid receptor)
• PPARα/γ/glucocorticoid receptor
• And PPARα/γ/interferon-α receptor
• Plus “jump starter” for therapeutic induction of the tumor stress response/epigenetic modelling with metronomic low dose chemotherapy or azacitidine
• Specific editing of NOA bottlenecks in tumors, e.g., mTOR

The systematization of NOA protein tools is often based on specific aspects, such as the description of transcriptional, neuronal, stress response or clinically relevant NOAs (Nagel et al., 2016; Di Marco et al., 2024; Bradner et al., 2017). Schematic NOA classification systems fail to specify that NOA proteins are fully functional tools in transcriptionally regulated circuitries that can be reprogrammed, despite being transcriptionally addicted by oncogenic aberrations (Figure 2). Thus, by coping with cancer hallmarks and stress responses, NOA proteins remain communicatively linked to establish the tumor phenotype (Weinstein and Joe, 2008; Hjaltelin et al., 2019; Weinstein, 2002).

Comparative studies of tumor tissue editing therapies show that critical functions to “normalize” cancer hallmarks and tumor stress responses are preserved by the transcriptomic landscape of tumor tissue and can be regulatory restored by specific targeting of nuclear/cytokine receptor NOAs with receptor agonists that exploit reprogramming of the NOA tool available for cancer hallmarks and stress responses. M-CRAC, endogenous stress response and tumor tissue editing approaches reorganize NOA circuitries (Figures 1, 2) (Harrer et al., 2023b; Harrer et al., 2025).

A well-established systems pharmacological approach, the transcriptional reprogramming of hormone-dependent cancers with receptor antagonists, has now been complemented by a combined targeting of nuclear/cytokine receptors with receptor agonists to restore tumor tissue functions via the transcriptomes of tumor cell compartments on the background of a therapeutically stress-activated or epigenetically reprogrammed tumor tissue on basis of low-dose metronomic chemotherapy or epigenetic modelling, e.g., with azacitidine (Figure 2; Table 3).

**TABLE 3:** Tumor tissue editing, phase I/II trials.

Tumor tissue editing, Phase I/II trials										
Metastatic relapsed/refractory neoplasia	Pioglitazone	Pioglitazone Interferon-α	Pioglitazone Dexamethasone	Pioglitazone All-trans retinoic acid	Small molecule	“Jump-start” therapy	Best response	Reprogramming of cancer hallmarks	Accompanying treatment effects	References
Angiosarcoma	+	​	​	​	COX-2 inhibitor	LDMC	cCR	-	Long-term cCR > 18 years	Vogt et al. (2003)
Melanoma Randomized	+	​	​	​	COX-2 inhibitor	LDMC	CR	Inflammation control	PPARγ expression prognostic marker	Reichle et al. (2007a)
Hepatocellular carcinoma	+	​	​	​	COX-2 inhibitor	LDMC	PR	-	-	Heudobler et al. (2021)
Cholangiocellular carcinoma	+	​	​	​	COX-2 inhibitor	LDMC	cCR	-	cCR > 5 years, lost of follow-up	​
Gastric cancer randomized	+	​	​	​	COX-2 inhibitor	LDMC	PR	-	No impact of pioglitazone in randomized trial	Reichle (2010)
High grade glioma	+	​	​	​	COX-2 inhibitor	LDMC	SD	-	-	Hau et al. (2007)
NSCLC randomized	+	​	​	​	COX-2 inhibitor clarithromycin	LDMC	PR	-	Sensitization to immune checkpoint inhibitor?	Heudobler et al. (2021)
Renal clear cell carcinoma	+	​	​	​	COX-2 inhibitor	LDMC	SD	No inflammation control	-	Hart et al. (2015)
Renal clear cell carcinoma	​	+	​	​	COX-2 inhibitor	LDMC	cCR	Inflammation control	Long-term cCR > 10 years	Reichle et al. (2007b)
Langerhans cell histiocytosis	​	+	​	​	COX-2 inhibitor	LDMC	cCR	Inflammation control	Long-term cCR > 6 years	Harrer et al. (2022)
Langerhans cell histiocytosis	​	​	+	​	COX-2 inhibitor	LDMC	cCR	Inflammation control	Long-term cCR > 10 years	Harrer et al. (2022)
Hodgkin’s lymphoma	​	​	+	​	COX-2 inhibitor everolimus	LDMC	cCR	Inflammation controlImmune response	Edited mTOR addictionLong-term cCR >10 years	Lüke et al. (2021)
Multiple myeloma	​	​	+	​	Lenalidomide	LDMC	CR	Inflammation control	Resolution of IMiD resistance	Heudobler et al. (2019a)
Castration-resistant prostate cancer	​	​	+	​	COX-2 inhibitor Imatinib	LDMC	PR	Immune responseInflammation control	Regain of hormone sensitivityNo impact of imatinib	Walter et al. (2010); Vogelhuber et al. (2015)
Acute myelocytic leukemia	​	​	​	+	-	Azacitidine	CR	Differentiation	Leukemic neutrophils	Heudobler et al. (2024a)

PPARα/γ, peroxisome proliferator activated receptor α/γ; RARs, retinoid receptors; M-CRAC, post-therapy metastatic spread, cancer repopulation and acquired tumor cell resistance and tumor cell heterogeneity; mTOR, mammalian target of rapamycin; COX-2, cyclooxygenase-2; LDMC, low dose metronomic chemotherapy with capecitabine or treosulfan or trofosfamide; SD, stable disease; PR, partial remission; CR, complete remission; cCR, continuous complete remission; ys, years with ongoing best reponse.

### Concerted use of only synergistically active drugs to redirect addicted tumor systems in a targeted manner: systems pharmacology

For the establishment of network-based systems pharmacology to target tumor tissue addictions, the analysis of transcriptional regulators for NOA circuitries would be of central interest regarding how systems-therapeutically accessible cancer hallmarks and tumor stress responses, can be reprogrammed or deregulated to attack tumor viability and induce tumor cell death (Figure 1; Table 4).

**TABLE 4:** Classification of tumor tissue addictions and therapeutically addressed addiction clusters.

Classification of tumor tissue addictions and therapeutically addressed addiction clusters
Identical tumor tissue addictions across tumor histologies (NOA circuitries coping with tumor-associated inflammation)
• cCR with identical editing schedule in multisystem Langerhans cell histiocytosis (mLCH), renal clear cell carcinoma (RCCC)
Links to other tumor-associated addiction clusters
• Tissue editing of NOAs
• Castration-resistant prostate cancer: Regain of hormone sensitivity, sustained PR after therapy discontinuation
• Multiple myeloma (MM): resolution of IMiD resistance
• Edited mTOR addiction in Hodgkin’s lymphoma
• Edited cachexia in melanoma
In case of CR and cCR identical tumor tissue addictions (transcriptionally regulated NOA networks) at metastatic sites
• Angiosarcoma, Cholangiocellular carcinoma, mLCH, Hodgkin’s lymphoma (HL), RCCC
• Melanoma, MM, acute myelocytic leukemia (AML)
Targeting of tumor-specific clusters of tumor tissue addictions
• Defined by receptor-triggered transcription factors
• Pioglitazone, all-trans retinoic acid
• CR in Non-promyelocytic acute myelocytic leukemia (non-PML AML)
• Pioglitazone, dexamethasone
• CR: mLCH, HL, MM; PR in castration-resistant prostate cancer (CRPC)
• Pioglitazone, interferon-α
• cCR: mLCH, RCCC
• Pioglitazone:
• CR, cCR: Melanoma (randomized ± pioglitazone), hepatocellular carcinoma (histologically PR), cholangiocellular carcinoma
• PR: Non-small cell lung cancer (NSCLC) (randomized versus Pembrolizumab), hepatocellular carcinoma
• PR: but no additional impact of pioglitazone in gastric cancer
• SD: High grade glioma
• Defined according to reprogramming of cancer hallmarks (clinically proven)
• Differentiation
• non-PML AML
• Inflammation control
• mLCH, HL, MM, RCCC, CRPC, non-PML AML
• RCCC: only with inflammation control delayed cCR
• Improvement of immunosurveillance
• CRPC, HL, non-PML AML

**FIGURE 3:**
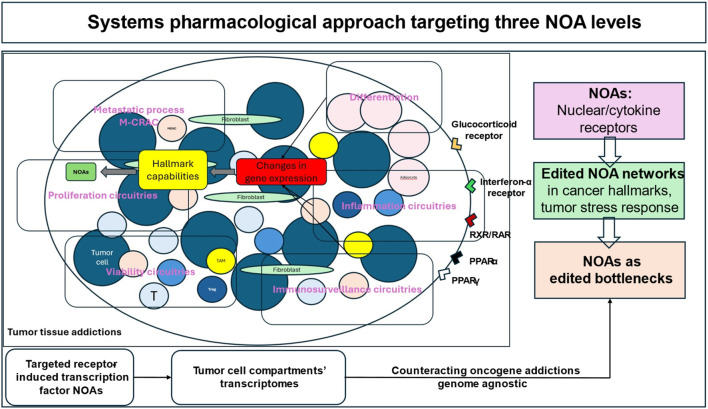
Systems pharmacological approach targeting three NOA levels via transcriptional modulation. The conceptual models presented here suggest that NOA-centered systems (circuitries) are interconnected in a modular way and respond differently to receptor-triggered transcriptional modulation by using receptor agonists such as pioglitazone. This results in various biological outcomes, such as controlling inflammation, enhancing immunosurveillance, inducing differentiation, and reprogramming tumor metabolism. These outcomes are biologically weighted differently, but are clinically uniform, with the possibility of long-term tumor control, objective response, CR or cCR induction. Shifting from a molecular to a modular approach to reprogramming therapy blurs the distinction between molecular programs consisting of pathways or transcription factors that are central to the activity of a specific histological tumor type. This is particularly pertinent given that activity profiles may be genome-agnostic.

A recurrent catalog of gene modules among tumors, correlated with recurrent cancer cell states, e.g., stress and interferon response or epithelial-mesenchymal transition, was identified that could explain therapeutically classifiable NOA circuitries dealing with cancer hallmarks/stress responses (Figure 3) (Barkley et al., 2022).

However, there is a lack of preclinical data on how cancer hallmarks and their activity profiles can be transcriptionally redirected for therapeutic purposes, how endogenous oncogene-addicted transcriptional control functions to maintain tumor tissue addictions, and which tumor-specific addictions to clustered stress responses/cancer hallmarks are transcriptionally targetable vulnerabilities (Figure 3) (Hjaltelin et al., 2019; Solimini et al., 2007).

Tumor tissue editing, as a systems pharmacology approach, has now pioneered the reprogramming of addicted transcriptome landscapes in tumor tissues by targeting selected receptor-induced transcription factor NOAs with receptor agonists to restore “normalized” NOA circuitries that resume activity among cancer hallmarks and stress responses by unleashing therapeutic phenotypic plasticity, differentiation, inflammation control, improved immune surveillance and pioglitazone-deregulated addictive energy metabolism, in patients with heavily pre-treated metastatic r/r neoplasias (Figure 3) (Gottfried et al., 2011).

In addition, individually edited NOAs have been identified as outcome-determining bottlenecks when targeted with the corresponding NOA inhibitor, e.g., everolimus (Figure 3) (Harrer et al., 2025).

The clinically observed reprogramming profiles of targeted cancer hallmarks refer to those hallmarks that are co-organized by tumor cells and neighboring stromal cells, i.e., inflammation control, enhancement of immune surveillance, addictive tumor metabolism, and therapeutic promotion of phenotypic plasticity, which is best explained by differentiation induction in r/r non-promyelocytic (non-PML) AML (Sibai et al., 2022; Heudobler et al., 2024a; Meier-Menches et al., 2022).

**FIGURE 4:**
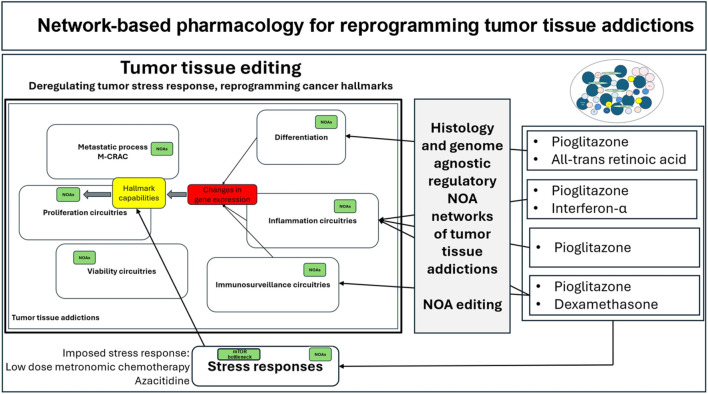
Network-based pharmacology for reprogramming tumor tissue addictions. Circuitries consist of modularly organized NOAs and are therapeutically accessible via systems pharmacological approaches such as tumor tissue editing.

The primary systemic pharmacological goal of tumor tissue editing approaches is to gain access to retained and pharmacologically retrievable circuitries in cancer hallmarks and stress responses via synergistically linked activities of receptor agonist-induced transcription factors, in the context of therapeutically driven stress response and epigenetic modeling. As shown, nuclear/cytokine receptor agonists encounter classifiable clusters of tumor tissue addictions and deregulate/reprogram tumor stress responses/cancer hallmarks via NOAs and NOA circuitries (Figure 4).

Therapeutic agonists of receptor-induced transcription factor NOAs target the same nuclear/cytokine receptors that oncogene addictions use to maintain tumor phenotypes. Reprogramming with receptor agonists results in measurable changes in cancer hallmarks and tumor identity, tumor cell death, and long-term tumor control of r/r hematologic neoplasms, cancers, and sarcomas (Figure 4) (Zhao and Iyengar, 2012; Harrer et al., 2025; Heudobler et al., 2018b; Nogales et al., 2022).

### Tumor plasticity, simultaneously therapeutic target and prerequisite for developing drug resistance

Non-PML AML, treated via non-oncogene addiction targets, can be used as a model to distinguish between AML plasticity that is therapeutically exploited, and therapeutically mediated AML plasticity that is induced by systemic AML therapies leading to drug resistance, e.g., to azacitidine (Heudobler et al., 2024a; Thomas et al., 2015; Kattner et al., 2020; Heudobler et al., 2018a).

In all three studied AML patients who developed azacitidine resistance during the immediate azacitidine monotherapy, resistance could be overcome by triple transcriptional modulation involving pioglitazone and ATRA, as indicated by CR induction in two patients and objective response in one patient (Heudobler et al., 2024a; Thomas et al., 2015; Heudobler et al., 2018a; Kattner et al., 2020). Triple transcriptional modulation plus azacitidine reprograms tumor-promoting hallmarks via differentiation induction and inflammation control by implementing reciprocal activity in NOA driven circuitries. The similar resistance resolving phenomenon has been observed in cases of IMiD resistance in multiple myeloma and imatinib failure to induce complete molecular remission in CML (Figure 1) (Heudobler et al., 2019a; Rousselot et al., 2017; Prost et al., 2015).

### Tumor tissue editing therapy: transcriptionally editing tumor tissue homeostasis and identity via addicted NOA circuitries

Receptor NOAs have established a transcriptional-based systems coherence, maintenance and reprogramming capabilities of NOA circuitries, thereby covering cancer hallmarks/tumor stress responses, as summarized by the following clinical observations: (1) Tumor tissue editing has therapeutically unlocked the tumor phenotypes by targeted reprogramming of cancer hallmarks with distinct combinations of nuclear/cytokine receptor agonists (Figure 4). (2) The 15 phase I/II studies demonstrate that selected, highly specific recombinations of receptor-triggered transcriptional modulators are necessary for reprogramming distinct patterns of cancer hallmarks (Table 3). (3) Nuclear/cytokine receptor agonists induce pivotal tumor responses up to CR and cCR in r/r neoplasms. (4) Tumor tissue editing resolves or attenuates M-CRAC in metastatic r/r neoplasia (Figure 1).

M-CRAC further illustrates that the NOA proteome is endogenously cross-linked by transcriptional programs that can be severely challenged and adapted via stress responses to pulsed systemic therapies (Hjaltelin et al., 2019; Harrer et al., 2023b). Endogenously transcriptionally regulated clustered NOA circuitries of cancer hallmarks and tumor stress responses implement timely tumor stress relief, even in response to severe therapy-induced stress, to rescue tumor viability (Figure 1).

Upon systemic pulsed tumor therapy, tumor stress responses are uniquely rewired across different tumor histologies to organize sufficient tumor stress relief (Hjaltelin et al., 2019). Thus, histologically different types of cancer may have stress-activated mechanisms in common, while stress responses may be differentially organized (Hjaltelin et al., 2019).

Tumor tissue editing as a targeted, metronomic, transcription-modifying therapy is characterized by the ability to prevent sufficient stress relief in tumor tissue and to reprogram cell-type specific regulatory circuitries within transcriptionally targeted cancer hallmarks (Figure 4). Thus, tumor tissue editing leads to profound changes in tumor tissue identity and homeostatic balance. The directly clinically observed reprogrammed cancer hallmarks were inflammation control, enhanced immune surveillance, and differentiation (Table 4) (Papi et al., 2014; Glass and Ogawa, 2006; Dicitore et al., 2014; Klobuch et al., 2018; Tilg et al., 1995; Senft and Ronai, 2016; Reichle, 2010; Bradner et al., 2017; Heudobler et al., 2024a; Heudobler et al., 2024b; Vogt et al., 2006; Lüke et al., 2021).

Previous NOA classifications have failed to highlight large-scale receptor-induced transcription factors that have emerged as targetable NOAs, dramatically altering NOA circuitries and therapeutically unlocking tumor phenotypes (Figure 4).

Targeting selected clusters of receptor NOAs involved in vulnerable tumor tissue addictions with drugs that have little or no monoactivity, but regulatory synergistic activity, is sufficient to reprogram cancer hallmarks, deregulate tumor stress responses, and edit individual NOAs for therapeutic purposes (Figures 1, 2; Table 3). In the presented studies, the configuration of selected cancer hallmarks available for transcriptional reprogramming is decisively supported by the adjacent cancer microenvironment, i.e., inflammation, immune response, tumor metabolism, energy metabolism and uncontrolled proliferation (Table 3) (Sibai et al., 2022; Heudobler et al., 2024a; Lüke et al., 2021; Harrer et al., 2022; Reichle et al., 2007a; Reichle et al., 2007b; Vogt et al., 2003; Walter et al., 2017; Reichle et al., 2010; Reichle et al., 2009; Hau et al., 2007; Heudobler et al., 2021; Heudobler et al., 2019a; Walter et al., 2010; Heudobler et al., 2018b; Hart et al., 2015; Vogelhuber et al., 2015).

Metronomic (very) low dose chemotherapy or epigenetic regulation, e.g., with azacitidine in non-PML AML, are essential activators of clinically relevant transcriptional modulation (Tables 3 and 4; Figure 3) (Harrer et al., 2023b). Targeting receptor-driven transcriptional regulators without metronomic low dose chemotherapy or azacitidine is ineffective in the setting of metastatic r/r neoplasia (Table 3). Tumor tissue editing reassigns low dose metronomic chemotherapy to a completely novel task, namely, to induce stress responses in tumor tissues. The choice of cytotoxic agent seems to play a minor role in extremely low dose metronomic chemotherapy. Among the alkylating agents, trofosfamide and treosulfan are less emetogenic than cyclophosphamide (Harrer et al., 2023b).

The known biological activities of the nuclear/cytokine receptor agonists used are indicative of broad reprogramming and deregulatory activities (Heudobler et al., 2024b). The transcriptomic landscapes in tumor cell compartments that operate tumor-specific tissue addictions, as shown, retain unlockable reprogramming profiles of cancer hallmarks.

Cyclooxygenase-2 (COX-2) inhibitors synergize with pioglitazone, contribute to NOA editing in r/r HL and does not augment clinical activity of interferon-α in r/r RCCC (Gehrmann et al., 2004; Schwandt et al., 2011; Harrer et al., 2025). The clinical effect of COX-2 inhibitors in many schedules seems to be supporting, as pioglitazone is the outcome determining substance in a randomized trial metronomic chemotherapy and COX-2 inhibitor plus/minus pioglitazone in metastatic r/r melanoma and the combination of receptor agonists used for tumor tissue editing classifies the editing of NOA circuitries coping with cancer hallmarks (Reichle et al., 2007b; Zelenay et al., 2015; Chan et al., 2007; Yang et al., 2025; Harrer et al., 2023b).

**FIGURE 5:**
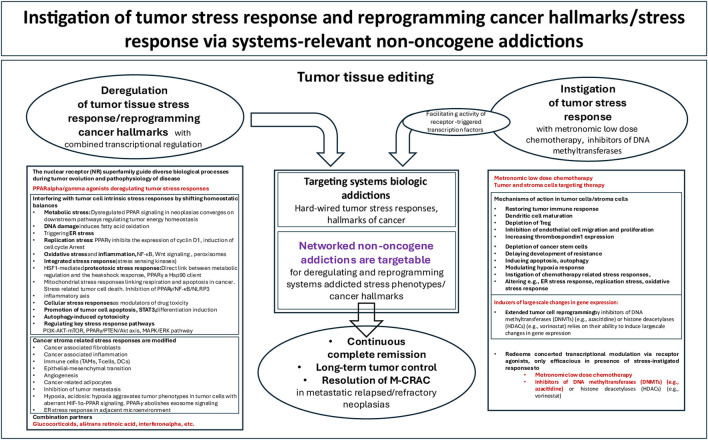
Metronomic chemotherapy or azacitidine instigate tumor stress responses in addition to endogenous tumor stress responses given by genetic aberrations. Continuous maintenance of therapeutically induced stress responses by low dose metronomic chemotherapy, epigenetic modulators or targeted therapies, e.g., MEK inhibitors, is prerequisite for the activity of combined transcriptional modulation. Instigation and deregulation of tumor stress response via systems-relevant non-oncogene addictions is organized in NOA circuitries.

Known patterns of response to metronomic low-dose chemotherapy add important biological effects (Cornice et al., 2024; Heudobler et al., 2018b) (Figure 5). Metronomic chemotherapy and low-dose azacitidine have well-studied pleiotropic activity profiles, including a T-cell-specific immune response in animal models and “jump-starting” activity in tumor tissue editing trials (Figure 4) (Ge et al., 2012; Harrer et al., 2023b). A continuous tumor stress response is necessary to facilitate the “jump-start” of the tumor systems’ transcriptional editing (Lüke et al., 2022). Intolerance to very low doses of metronomic chemotherapy was an exclusion criterion in all studies, as transcription modulation is ineffective without mild “cytotoxicity” as a backup. The same applies to the combination of transcriptional modulators with targeted therapies (Ben-Yishay et al., 2024; Prost et al., 2015; Plumber et al., 2024; Heudobler et al., 2019a).

#### Stress response to metronomic chemotherapy

Metronomic chemotherapy induces cell stress responses and DNA repair, as well as activation of cell death, which facilitates a T-cell-specific immune response. This response is important for upstream regulators such as toll-like receptors, interferons and peroxisome proliferator-activated receptor (PPAR) signaling pathways, which contribute to tumor immunity. Metronomic chemotherapy also inhibits hypoxia-inducible factor 1 alpha (HIF-1α) and MAP kinases, which are often involved in promoting tumor growth. Chemotherapeutic stress increases angiogenesis via NF-κB-dependent Akt expression and VEGF (Meng et al., 2007).

NFκB is intrinsically regulated in many tumors particularly in metastatic and pre-treated tumors, like those with downregulated STAT3 (McCarty and Block, 2006). As PPAR signaling interferes with the endoplasmic reticulum (ER) stress response, the accumulation of unfolded or misfolded proteins in the ER is causally involved in ER stress. The unfolded protein response (UPR) is then activated and may be involved in PPAR activities (He Jiang et al., 2024). The crosstalk between PPAR signaling and the WNT/β-catenin pathway, chronic tumor-associated inflammation, and oxidative stress is well established (Vallée and Lecarpentier, 2018).

#### Limitations of the summarized clinical trials

The conclusions drawn from clinical trials on NOA-based systems pharmacology are based on three randomized phase II trials involving patients with metastatic r/r melanoma, NSCLC (versus immune checkpoint inhibitor) and gastric cancer (± pioglitazone). All the other trials were single-arm phase I/II trials, including a phase I trial in acute myelocytic leukemia (AML). These trials principally demonstrate that NOA-based therapies offer significant potential for meaningful therapeutic responses, even in cases of recurrent or refractory metastatic neoplasms, including hematological malignancies, cancers, and sarcomas (Table 3) (Harrer et al., 2023b).

The main limitation of the trial designs is the small number of patients included. Consequently, the trials cannot determine the frequency of responses to NOA-based systems pharmacology in single neoplasias. However, they do demonstrate the effectiveness of tumor tissue editing in histologically quite different r/r metastatic neoplasias and in case of non-PML AML in a genetically highly heterogeneous disease (Table 3).

### Off-target effects of tumor tissue editing techniques

In rare cases, clinically relevant off-target transcriptional effects occurred. In less than 5% of patients, therapy had to be discontinued due to the tumor tissue editing approach used. These favorable toxicity data were due to scheduled dose reductions for metronomic chemotherapy and the reduced frontline dose of azacitidine in AML, compared to the approved standard dose (Heudobler et al., 2024a). Scheduled dose reductions were used if off-target effects upon transcriptional modulation or chemotoxicity occurred. Pioglitazone may induce edema, which is dose-dependent in most patients, and may lower blood glucose levels in diabetic patients, necessitating adjustments to antidiabetic therapy. COX-2 inhibitors plus pioglitazone may induce mild renal insufficiency, which can be alleviated by discontinuing the COX-2 inhibitor. Interferon-α was primarily used at a low dose. Thus, interferon-α did not have to be discontinued due to neurological or psychiatric problems (Harrer et al., 2023b).

Off-target effects of classic targeted therapies, such as mTOR inhibitors have to be considered, e.g., pneumonitis following mTOR inhibition with everolimus (n = 1 patient with Hodgkin’s lymphoma) (Harrer et al., 2025).

### Receptor-regulated transcription factor NOAs deregulate or reprogram selected clusters of NOA circuitries involved in cancer hallmarks

The combinations of agonists targeting receptor-triggered transcription factors have been chosen on the basis of their mostly modest but well-known clinical activity: interferon-α has activity at high doses in renal clear cell carcinoma (RCCC), everolimus in Hodgkin’s lymphoma and glucocorticoids in hematological neoplasms (Aldin et al., 2023; Guarini et al., 2012; Qualls et al., 2025). Pioglitazone is the connecting link, as peroxisome proliferator-activated receptor (PPAR) agonists are frequently expressed in malignant cells, particularly in metastatic diseases (Reichle 2010). Tumor proteomic profiling has not yet been systematically performed with respect to receptor-triggered transcription factors and corresponding down-stream transcription factors. Proteomic platforms, as shown, can be used to test, e.g., for AML “normalization,” e.g., differentiation *in vitro* (Meier-Menches et al., 2022).

#### Peroxisome proliferator-activated receptors α/γ (PPARα/γ)

In all treatment studies presented, the PPARα/γ agonist pioglitazone provided fundamental support through transcriptional activation of PPARα/γ-mediated target genes: the addition of pioglitazone to low-dose metronomic chemotherapy could significantly improve outcome in r/r metastatic melanoma in a randomized comparison (Ranjan et al., 2024; Harrer et al., 2023a; Reichle et al., 2007b) (Table 3).

Metabolic reprogramming of cellular energy balance is considered a cross-cancer hallmark; therefore, this hallmark appears to be a prerequisite for all neoplastic cells and places PPARα/γ agonists at the center of therapeutic efforts to reprogram stromal coregulated cancer hallmarks in many neoplasias (Table 4) (Sibai et al., 2022; Harrer et al., 2023a; Vallée and Lecarpentier, 2018; Gottfried et al., 2011).

PPARα/γ are known as ligand-induced transcription factors with a broad modularly guided activity profile that link lipid metabolism to cellular signaling via natural ligands, the peroxisomes, in a cell type-dependent manner (Reichle and Hildebrandt 2009) (Figure 3) (Schrader and Fahimi, 2006; Harrer et al., 2023a). The activity profile of PPARα/γ is specified by the interaction with other nuclear/cytokine receptors (Kökény et al., 2021; Liu et al., 2003). Physiological interactions are used with the retinoid receptor, RAR, and glucocorticoid receptor (Klobuch et al., 2018; Glass and Ogawa, 2006).

In cancer, the mono-therapeutic effects of approved ligands of receptor-induced transcription factors are negligible. *In vitro* data, however, shows a close link between impressive effects on stress response pathways and cross-linked cancer hallmarks (Figure 5).

PPARγ expression is dependent on tumor histology and stage, with expression often increased in metastatic tumors revealing that PPARγ may be stage-dependently involved in promoting tumor systems addictions (Meyer et al., 2009; Meyer et al., 2010; Schöckel et al., 2024). The trials presented recruited patients with metastatic r/r neoplasia only.

The targeted receptor-activated transcription factors, PPARα/γ, retinoic acid receptor (RAR), glucocorticoid receptor or interferon-α receptor, are vital receptors and ubiquitously distributed in the cellular compartments of tumors (Hannigan et al., 1986; Meyer et al., 2010).

Therapeutically, PPARα/γ agonists deregulate multiple tumor stress responses that are highly addicted in tumors, such as metabolic, proteotoxic, mitotic, oxidative, DNA damage stress and linked to stress responses, upregulated autophagy (Watanabe et al., 2023; Vallée and Lecarpentier, 2018; Gehrmann et al., 2004) (Figure 5). In addition, PPARα/γ agonists are involved in reprogramming a broad pattern of cancer hallmarks, immunosurveillance, inflammation, proliferation, tumor metabolism, angiogenesis, invasion, and systemic host responses to the tumor, such as cachexia (Muqaku et al., 2017; Harrer et al., 2023a).

Recombinations of agonists of receptor-triggered transcription factors, PPARα/γ/RAR; PPARα/γ/glucocorticoid receptor; and PPARα/γ/interferon-α receptor, as shown, can be adapted to the individually established type of genome-agnostic regulatory hard-wired NOA circuitries (Tables 1 and 4). Individual counterregulation of cancer hallmarks, deregulation of tumor stress responses and NOA editing are a typical feature of tumor tissue editing therapies, as shown. Pre-therapeutic diagnostic parameters are not currently available for routine selection of nuclear/cytokine receptor agonists (Heudobler et al., 2024a; Harrer et al., 2025) (Figure 4).

#### Interferon-α

The activity profile of interferon-α in tumor tissues is determined by the frequent expression of interferon responses across tumor types and adjacent stromal cells, and that responses are coregulated. Type I interferons directly regulate the transcription of more than 100 downstream genes (Borden, 2019). Interferon responses seem to be a prerequisite for tumorigenesis (Barkley et al., 2022; Cheon et al., 2023; Vidal, 2020). Described ambiguous interferon-I tumor responses appear excluded by pioglitazone’s coregulatory activity (Cheon et al., 2023).

#### All-trans retinoic acid

Preclinical data show that the differentiation-promoting combination of ATRA and pioglitazone is only active with simultaneous epigenetic modulation using azacitidine as a “jump start” (Klobuch et al., 2018).

#### Dexamethasone

Peroxisome proliferator-activated receptors (PPARs) and glucocorticoid receptors control proinflammatory and metabolic programs and gene expression in neoplastic cells, macrophages and lymphocytes and regulate innate and adaptive immunity (Glass and Ogawa, 2006).

#### Stroma effects promoted by transcriptional modulation

The effects of pioglitazone on tumor stroma cells have been recently described in detail (Harrer et al., 2023a). PPARγ agonists demonstrate potent immunoregulatory effects on the entire T-cell repertoire, as well as on macrophages, dendritic cells and their intercellular behavior. Adipocytes are additionally decisively regulated by PPARγ agonists (Hernandez-Quiles et al., 2021). PPARγ agonists also regulate the expression of genes involved in lipid metabolism and transport. These include the class B scavenger receptor CD36, as well as FABP4, LXRA and PGAR, in all T-cell and macrophage subtypes. The reprogrammed lipid metabolism may also reactivate effector T cells to promote immunological tumor responses (Harrer et al., 2023a). PPARγ agonists may improve the response to immune checkpoint inhibitors (Heudobler et al., 2021; Reuthner et al., 2024; Zhan et al., 2022).

The interaction between PPARα/γ agonists and stress responses can be best illustrated by examining the effects of pioglitazone on the mTOR pathway in the context of tumor tissue editing therapies, as mTOR acts as an interface for many stress response pathways. In particular, tumor-associated inflammation and the oxidative stress response are controlled by PPARγ (Harrer et al., 2025).

### Targeted combinations of receptor-induced transcription factors

To differentially target inflammation addiction the following receptor combinations were selected for dual/triple transcriptional modulation with the PPARα/γ agonist pioglitazone, PPARα/γ/glucocorticoid receptor agonists or PPARα/γ/interferon-α receptor agonists (Table 3).

The use of cancer hallmarks that are co-regulated by the microenvironment as targetable tumor tissue addictions offers important advantages. Microenvironment-targeted tumor cell behaviors can be transcriptionally deregulated in critical ways, while stromal conditions can be stimulated to prevent tumor cell growth (Xiao et al., 2024; Hendrix et al., 2007; Sibai et al., 2022; Ge et al., 2012). Even metastatic tumors conditioned with M-CRAC that are refractory or recurrent can be sufficiently targeted by therapeutically involving tumor and stromal cells to be controlled in the long term (Vitale et al., 2019; Harrer et al., 2023b).

Tumor tissue editing approaches also successfully use the metronomic principle for the application of receptor-triggered transcription factor agonists. The large-scale configuration of clustering tumor tissue addictions determines the respective therapeutically suitable recombination of receptor agonists (Table 3).

Inflammation as a hallmark of cancer is transcriptionally accessible differently depending on the type of inflammatory addiction, either with pioglitazone alone as a PPARα/γ agonist (angiosarcoma, melanoma, glioblastoma, cholangiocellular carcinoma, hepatocellular carcinoma and gastric cancer) (Vogt et al., 2003; Reichle et al., 2007b; Hau et al., 2007; Walter et al., 2017; Reichle, 2010; Reichle et al., 2010; Reichle et al., 2009) or in the combination pioglitazone/interferon-α [renal clear cell carcinoma, multisystem Langerhans cell histiocytosis (mLCH)] (Reichle et al., 2007a; Harrer et al., 2022), or pioglitazone/dexamethasone in mLCH, multiple myeloma (MM), castration-resistant prostate cancer (CRPC) (Harrer et al., 2022; Heudobler et al., 2019a; Vogelhuber et al., 2015; Walter et al., 2010; Hart et al., 2015; Prins et al., 2012), and pioglitazone/dexamethasone/everolimus in r/r Hodgkin’s lymphoma (HL) (Lüke et al., 2021; Harrer et al., 2025).

Measurement of serum C-reactive protein (CRP) in HL, renal clear cell carcinoma (RCCC), mLCH, and melanoma served as a follow-up marker of inflammatory response. CRP response correlated well with tumor shrinkage, but shrinkage was delayed in r/r RCCC (Hoefflin et al., 2020; Reichle et al., 2007a; Lüke et al., 2021; Harrer et al., 2022; Reichle et al., 2007b; Reichle and Vogt, 2008).

The combination of pioglitazone and all trans-retinoic acid is characterized by a strong differentiation induction in non-PML AML, even in AML with a complex aberrant karyotype but also in muscle invasive bladder cancer in animal models (Heudobler et al., 2024a; Thomas et al., 2015; Plumber et al., 2024).

Qualitatively completely different activity patterns depending on the respective co-modulator of pioglitazone, RAR, glucocorticoid receptor or interferon-alpha receptor are probably the reason that these receptors have not yet appeared as NOAs. Their clinically relevant interactions and the possibility of combinatorial specification of their activity profiles are therapeutically crucial for r/r metastatic neoplasias (Table 3).

### Transcriptionally edited NOAs specific to tumors

#### r/r HL tissue

Tumor-specific, transcriptionally altered NOAs emerge as targets for NOA inhibitors (Harrer et al., 2025; Neganova et al., 2021). Editing the mammalian target of rapamycin (mTOR) as a bottleneck in metastatic r/r HL with pioglitazone/dexamethasone demonstrates how NOAs, here mTOR, can become a critical rate-limiting target. According to trial summary statistics, treatment of edited r/r HL tissue with dose-adjusted everolimus enables long-term continuous complete remission in seven consecutive patients suffering from metastatic r/r Hodgkin’s lymphoma. Everolimus, an mTOR inhibitor, was a prerequisite for rescuing r/r Hodgkin’s lymphoma in edited Hodgkin tissue. Otherwise, everolimus has only a weak activity profile in r/r HL (Harrer et al., 2025).

With systems pharmacology approaches only, bottlenecks in the NOA pathways could be identified, e.g., the relevance of mTOR in r/r Hodgkin lymphoma. The mTOR bottleneck was proven in r/r Hodgkin’s lymphoma treated with lymphoma tissue editing techniques (Harrer et al., 2025). Further evaluation of the molecular mechanisms leading to mTOR editing is required.

Possible molecular mechanisms promoting Hodgkin’s lymphoma editing have been summarized in a recently published paper (Harrer et al., 2025). The AKT/mTOR signaling pathway is centrally located and activated by various stress response pathways.

This example illustrates how systems pharmacological tissue-engineering therapies create cutting edges by transcriptionally converging tumor stress responses in an mTOR-dependent manner. Everolimus critically deregulates HL stress responses, including the endoplasmic reticulum stress response (Harrer et al., 2025) (Table 5). The possibility of single NOA editing provides further therapeutically relevant insights into the mechanisms of action of tumor tissue editing therapies.

**TABLE 5:** Concerted targeting of non-oncogene addictions networks with tumor tissue editing techniques.

Systems pharmacological, tumor tissue editing approaches:
Pioglitazone, pioglitazone/dexamethasone, pioglitazone/interferon-α, pioglitazone/all-trans retinoic acid plus low dose metronomic chemotherapy or azacitidine
• Doses adapted to biomodulatory activity profile of the single drug
• Toxicity: Modest off-target effects
• Wide therapeutic window for tumor tissue editing approaches: Differential susceptibility to stress overload in tumors compared to normal tissues, and cancer hallmarks compared to normal organ-defined cell identity
• Concerted activity profile of agonists of receptor-triggered transcription factors is crucial for therapeutic applications
• Individual drugs have poor or no monoactivity
• Possibility of using the maximum tolerable dose of a small molecule to target edited bottleneck NOAs (mTOR with everolimus)
• Genome agnostic therapy: tumor systems dependent on type of NOA networks
• Targeting clustered NOAs with drug combinations
• Template for repurposing nuclear/cytokine receptor agonists as drugs

HL is an important example where tissue editing itself may not be sufficient to induce lymphoma cell death, in this case Sternberg-Reed cells, but HL editing offers pivotal therapeutically edited NOA targets, a therapeutic approach that may be generalizable (Zhu et al., 2022; Waldschmidt et al., 2025).

#### Castration-resistant prostate cancer (CRPC)

Following treatment of CRPC with tissue-editing approaches, two observations stand out. Long-term disease stabilization in PR is possible despite treatment discontinuation, indicating stable epigenetic reprogramming of the CRPC tissue (Liang et al., 2017), which is associated with a regain of hormone sensitivity (Hart et al., 2015). Tumor editing may specifically resolve M-CRAC phenotype in CRPC through restoration of hormone sensitivity with pioglitazone plus dexamethasone (Prins et al., 2012).

Both metronomic low-dose chemotherapy in the CRPC treatment regimen and azacitidine in the AML treatment regimen can induce stress response but also promote epigenetic reprogramming (Heudobler et al., 2024a; Hart et al., 2015).

#### r/r multiple myeloma (MM)

Editing in multiple myeloma (MM) may overcome IMiD resistance, allowing successful reinitiation of IMiD therapy. Editing could potentially restore the CRBN-HSP90 axis and thereby increase IMiD activity. Tumor metabolism significantly alters IMiD activity, and pioglitazone/dexamethasone likely counterregulate the addictive homeostatic balance of MM metabolism (Heudobler et al., 2019a; Heider et al., 2021).

#### r/r melanoma

In r/r melanoma, pioglitazone has been shown to reverse melanoma-associated cachexia. Reprogrammed platelets appear to be involved (Muqaku et al., 2017; Hart et al., 2016).

The observations in r/r MM, CRPC, r/r HL and r/r melanoma impressively demonstrate that cross-linked addictive homeostatic processes are modularly integrated in tumor systems, with key cancer hallmarks such as inflammation and reduced immune surveillance additionally involving stromal cell addictions.

In general, the clinical identification of edited tumor-specific NOA addictions is a high priority to improve the efficacy of NOA-targeted therapies, such as targeting mTOR in r/r HL and restoring sensitivity to tumor-specific NOAs, androgen receptor or IMiD (Heider et al., 2021; Hart et al., 2015).

Pivotal clinical responses by targeting individual NOAs suggest the presence of primarily endogenously edited NOAs within tumor tissue addictions, e.g., in immune checkpoint inhibitor-sensitive neoplasias (Klaeger et al., 2017; Zhu et al., 2022; Butterfield and Najjar, 2024).

### Tumor tissue expression of NOAs

NOA addiction circuitries have been clinically identified as potential targets for NOA systems pharmacology. These NOA systems circuitries are suggested to mediate clinical response to NOA-targeted systems pharmacological approaches.

However, given that NOA addiction circuitries are modular in nature, the definition of circuitries does not imply that identical systems pharmacologic approaches will target identical circuitries. Identical transcriptional modulators, such as pioglitazone, used within systems pharmacology approaches, induce an objective response or continuous complete remission (cCR) via differential biological effects affecting tumor-associated inflammation, immunosurveillance, differentiation and tumor metabolism (Harrer et al., 2023a). Furthermore, in animal models, pioglitazone contributes to the induction of unexpected biological outcomes, such as fatty degeneration of tumor cells or tumor stem cell toxicity, depending on the combination partners (MEK inhibitors or imatinib) (Prost et al., 2015; Ishay-Ronen and Christofori, 2019).

Modular NOA-directed therapy is characterized by the extent to which NOA circuitries that maintain hallmarks of cancer, stress responses and M-CRAC can be separated and recombined therapeutically by tumor tissue editing approaches for finally disrupting tumor resilience and viability (Figures 1, 3). Thus, tissue editing approaches reveal a wide range of potential systems pharmacology approaches involving NOAs, which remain largely unexplored.

Therapeutic modeling of addicted tumor systems with editing approaches is multi-faceted and far-reaching, as the therapeutic approach spans multiple biological scales, from molecular interactions to cell behavior to communicative tissue architecture/ecology (Sibai et al., 2022) (Figures 1, 3).

Tumor microenvironments provide histology-specific NOAs. These NOAs are communicatively integrated into hardwired NOA circuitries, as stromal NOA patterns can even characterize histological tumor subtypes (Lenz et al., 2008). Thus, NOA proteins are typically distributed in all cellular tumor compartments and are characteristically organized to maintain stroma coregulated cancer hallmarks (Solimini et al., 2007).

Tumor tissue editing involves three therapeutically relevant NOA levels that provide information about cancers’ addictive traits (Figure 3).

The transcriptionally targeted NOA proteome copes with tumor stress responses and cancer hallmarks (Figure 3) (Di Marco et al., 2024). In addition, NOAs are also present within the organ invasive front of tumors (Tetzlaff et al., 2025; Jeong et al., 2025; Chastney et al., 2025; Blazquez et al., 2022).

The clinical observations of inflammation control and reprogramming of the stroma coregulated cancer hallmarks (improved immune response) in metastatic r/r neoplasias reveal that tightly structured inflammation-associated NOA circuitries regulate tumor-associated inflammation in a classifiable manner, across cellular tumor compartments and independent of tumor type, via receptor-induced transcription modulation (Table 3).

Clinical data on the reprogramming of tumor-promoting hallmarks, with the potential for consecutive complete response (CR) and cCR induction in highly heterogeneous tumor histologies, supports the pharmacological possibility of comprehensively reorganizing NOA circuitries across histologically highly different tumor tissues in a modular manner. This is achieved using NOA-based systems pharmacology approaches, which include agonists of receptor-triggered transcription factors such as peroxisome proliferator-activated receptors (PPARα and γ), interferon (IFN)-α, retinoid receptors (RAR), and the glucocorticoid receptor (Figures 2, 4).

Specific transcriptional approaches, epigenetic (azacitidine) and tumor stress promoting mechanisms are the basis for substantial reprogramming of NOA based circuitries, a process that can be easily cytologically visualized in AML. In non-PML AML, neutrophils harboring acquired genetic aberrations in the blasts can be detected following successful differentiation inducing NOA triggered therapy (Thomas et al., 2015).

Furthermore, a steep serum CRP response in a series of quite different neoplasias may precede continuous complete remission, suggesting that NFκB is a central target of the therapeutically used transcriptional modulators (Reichle et al., 2007a). A little-known phenomenon of interferon-α is its massive CRP-suppressive activity in healthy individuals (Tilg et al., 1995).

Figure 4 highlights transcriptional regulators targeted by receptor-triggered pathways, the PI3K-AKT-mTOR and the PPARγ/PTEN/Akt axes and the MAPK/ERK pathway (Glaviano et al., 2023).

Potential transcriptionally accessible NOA targets operating in NOA circuitries to control inflammation could be NF-kB, IL-6, COX-2, STAT3, VEGF and corresponding receptors (VEGFRs), HSP70/HSP90, mTOR, downregulated PTEN, general tumor-associated stress responses and NOAs in stromal cells (Zheng et al., 2013; Teresi and Waite, 2008; Harrer et al., 2023a; Spella et al., 2023; Petroni et al., 2022).

The central regulation of the mTOR pathway by tumor tissue stress responses and the counterregulation by tumor tissue editing has been summarized in the context of NOA based editing therapy in r/r Hodgkin’s lymphomas (Harrer et al., 2025).

Oncogenic aberrations only define the (common) inflammatory addiction phenotype, and thus the specific transcriptional accessibility. However, the oncogenic background does not affect the fundamental possibility of accessing NOA circuitries integrated in cancer hallmarks via tumor tissue editing techniques.

These findings greatly simplify the therapeutic targeting of cancer hallmarks using systems pharmacology approaches. However, the system-relevant molecular mechanisms remain to be elucidated.

In the future, diagnostics on circuitries that maintain NOA addiction and corresponding regulatory transcription factors will have a tremendous impact on the design of systems pharmacological therapy approaches with tumor tissue editing techniques.

### Systems therapeutic accessibility of NOA circuitries

Considering the histological and genomic diversity of recurrent and refractory metastatic neoplasias included in multiple clinical trials (Table 3), as well as the unique responses to NOA-based tumor tissue editing approaches ranging from long-term tumor control, objective response, CR to cCR, it appears that NOA circuitries are accessible across many different histological tumor types. However, the design of tumor tissue editing approaches must be adapted to the NOA circuitries and their modular links, as shown for r/r RCCC and mLCH (Table 3).

Gastric cancer was the only histology that did not significantly respond to pioglitazone in a randomized phase II trial among 15 different trials for highly varying tumor histologies, including cancers, sarcomas, and hematological neoplasias. Gastric cancer cells express PPARγ (Ezzeddini et al., 2021; Reichle, 2010). This suggests that modular circuitries may be resistant to PPARγ agonists in gastric cancer. The principal possibility of successfully adapting transcriptionally active combination therapies in a genome-agnostic way gives rise to the hope that differential combinations of agonists of receptor-triggered transcription factors may solve the gastric cancer problem.

Complete remissions could not be observed in r/r high-grade gliomas, hepatocellular carcinomas (one clinical remission histologically not confirmed), gastric cancer or NSCLC (Table 3). For using tumor tissue editing approaches as template for overcoming M-CRAC, NOA circuitries have to be studied in detail on a molecular basis.

### Oncogenic events counteracted by transcriptional remodelling of NOA circuitries

In analogy to oncogenic events, the editing process transcriptionally reorganizes and substantially reprograms cancer hallmarks, stress responses and leads to novel protein-protein interactions, altered pathway activities, stromal composition and tumor-stroma interactions (Li et al., 2023; Reichle, 2013; Heudobler et al., 2024a; Thomas et al., 2015; Heudobler et al., 2018b; Califano, 2011; Reichle and Hildebrandt, 2009; Hjaltelin et al., 2019) (Table 3; Figure 1).

Thus, breaking the tumor tissue addictions with editing approaches, as shown in many tumor types, r/r cancers, sarcomas and hematological neoplasms, is accompanied by crucial changes in the cancer phenotype (Harrer et al., 2025; Heudobler et al., 2024a; Harrer et al., 2025; Harrer et al., 2022; Reichle et al., 2007a) (Table 5).

Transcriptional interventions in the context of tumor stress response and cancer hallmarks always involve multiple hallmark activities simultaneously, beyond those clinically observed (Table 4). Due to the “multi-tasking” activity profiles of transcriptionally active, editing agents, hallmarks with stromal involvement - inflammation, immune response, angiogenesis, invasion and metastasis, release of phenotypic plasticity, sustained proliferative signaling, and deregulation of cellular energetics - are simultaneously addressed (Harrer et al., 2023a; Sibai et al., 2022).

From a tissue processing perspective, it does not seem to matter much that hallmark activities are heterogeneous within tumors. Agonist combinations may be adapted respectively, to oncogenic altered hallmark addictions (Sibai et al., 2022; Harrer et al., 2025; Harrer et al., 2022).

Clinically successful reprogramming of NOA circuitries that configure cancer hallmarks is best evidenced by CR and cCR induction in r/r non-PML AML, HL, mLCH, RCCC, angiosarcoma, cholangiocellular carcinoma, and melanoma (Table 3) (Reichle et al., 2007a; Ugocsai et al., 2016; Vogt et al., 2006; Lüke et al., 2021). Differentiation induction of leukemic blasts to neutrophils changes the identity of AML tissue. The genome-agnostic activity of tissue editing is at best demonstrated in AML: complex aberrant cytogenetics does not exclude differentiation induction and CR (Klobuch et al., 2018; Heudobler et al., 2024a; Heudobler et al., 2018a; Thomas et al., 2015). CR after tumor tissue editing could be successfully consolidated with allogeneic blood stem cell transplantation in r/r AML, or relapses after allogeneic blood stem cell transplantation could be rescued in r/r HL and AML (Kattner et al., 2020; Heudobler et al., 2024a; Lüke et al., 2021).

Although, NOA circuitries connect oncogenic drivers at different tumor tissue levels, NOA circuitries provide an autonomous transcriptionally accessible target (Li et al., 2023) (Table 5). The ability of specifically selected agonist combinations of nuclear/cytokine receptors to reprogram cancer hallmarks, to deregulate stress responses or to target NOAs as therapeutically relevant bottlenecks impressively demonstrates that NOAs are organized in transcriptionally hardwired NOA circuitries (Ma and Kroemer, 2024; Kusaczuk et al., 2024).

### Common NOA circuitries for tumor-associated inflammation in a range of tumor histologies

Tumor-associated inflammation plays a critical cross-cancer role in the promotion of tumor growth and M-CRAC (Harrer et al., 2023b; Sibai et al., 2022). Inflammation-associated serum biomarkers, such as CRP, facilitate the identification of tumor-associated inflammation as an important cancer hallmark with strong activity in histologically diverse tumor types, particularly in advanced disease, and allow us to empirically specify potential nuclear/cytokine receptor agonist combinations (Table 3) (Hart et al., 2020).

Suppression of inflammation and therapy-induced stress responses in tumor tissue with receptor agonists directly affects additional stroma-coregulated cancer hallmarks, immunosurveillance, angiogenesis, extracellular matrix, tumor proliferation and M-CRAC (Sibai et al., 2022; Harrer et al., 2023b).

**FIGURE 6:**
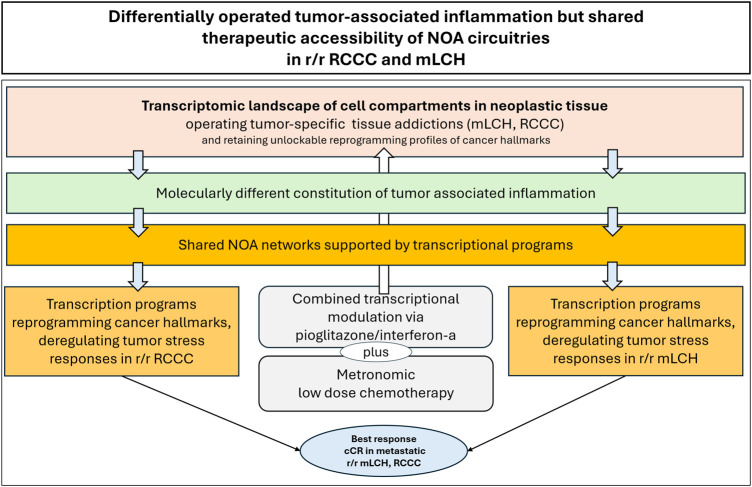
Differentially operated tumor-associated inflammation but shared therapeutic accessibility of modular organized NOA circuitries in r/r renal clear cell carcinoma (RCCC) and multisystem Langerhans cell histiocytosis (mLCH).

An important finding from the comparative analysis of tumor tissue editing data in metastatic r/r neoplasms is that histologically/genetically quite different tumors provide unique differential transcriptional access to their transcriptional landscape to reconfigure NOA circuitries that support tumor-associated inflammation and other stroma coregulated hallmarks, although tumor-associated inflammation, e.g., is profoundly differentially integrated in the tumor context in r/r mLCH and r/r RCCC, but outcome to pioglitazone/interferon-α with cCR may be identical (Table 3; Figure 6). A key difference in inflammation management between r/r mLCH and r/r RCCC is evident by the fact that RCCC cells directly release CRP into the serum, in addition to indirect CRP secreted by the liver (Jabs et al., 2005).

Pioglitazone is the common transcriptionally active ligand contributing to inflammation control in all presented editing trials, although experimental data demonstrate the extremely heterogeneous implementation of neoplasia-associated inflammation as a cancer hallmark (Jong et al., 2024). CR or cCR in quite different metastatic r/r neoplasias after selective reprogramming of tumor-associated inflammation supports the availability of ubiquitous, cross-tumor histology targetable transcriptional programs constituting classifiable NOA circuitries susceptible to pioglitazone plus/minus transcriptionally active combination partners (Table 4; Figure 4) (Hjaltelin et al., 2019; Harrer et al., 2022; Reichle et al., 2007a) (Figure 1; Table 3). These clinical observations were not predicted by current experimental data (Hjaltelin et al., 2019; Luo et al., 2009; Vervoort et al., 2022; Harrer et al., 2023b).

**FIGURE 7:**
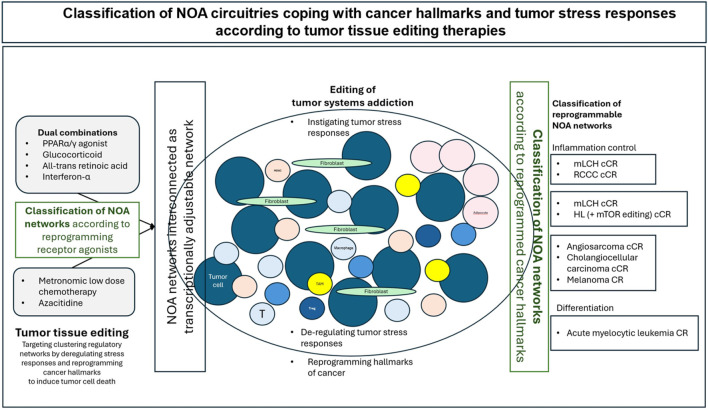
Currently, only a clinical classification of regulatory circuitries supporting cancer hallmarks and tumor stress responses is possible. NOA circuities are organized in modular operating NOA circuitries that are not related to single cell compartments but span tumor and adjacent stroma cells. The tumor cell compartment, including genetically aberrant cells and genetically normal cells contributing to tumor proliferation, constitutes major tumor hallmarks that are accessible for systems pharmacological therapy approaches.

The clinical data on the transcriptional processing of inflammation indicate that NOA circuitries associated with the hallmark inflammation represent important systems pharmacological targets for rescuing metastatic r/r neoplasias: the specificity of dual/triple transcriptional modulation targeting different transcriptionally regulated NOA circuitries is best illustrated in metastatic r/r neoplasias responding with CR and cCR, even if edited NOAs must be additionally targeted to achieve cCR, as shown for r/r HL (Table 4; Figure 7) (Reichle et al., 2007a; Harrer et al., 2022).

At least two different clusters of transcriptionally regulated NOA circuitries may be attributed to mLCH histology with respect to the hallmark inflammation, one responding to pioglitazone/dexamethasone with cCR, the other to pioglitazone/interferon-α (Figure 7) (Reichle et al., 2007a; Harrer et al., 2022).

Pioglitazone/interferon-α rescues pioglitazone/dexamethasone failure, even in the setting of disease-related bone marrow and liver failure (Harrer et al., 2022). The reason for a change of transcription modulators is probably quite clear. Addiction spans multiple biological scales, and thus, in the case of mLCH-associated liver and bone marrow failure, a maximized anti-inflammatory effect combined with interferon-α-linked immune modulation was significantly more effective (Harrer et al., 2022). The case highlights that tumor addiction involves not only addictions of the tumor tissue itself, but also reactions between tumor and host. This means that differential access to histologically identical neoplasms may also depend on the host-tumor interface.

Only after the addition of low-dose interferon-α to pioglitazone in a second study in metastatic r/r RCCC did objective responses occur and consecutive CR and cCR (Tupikowski et al., 2015; Reichle et al., 2007a). Outcome data in metastatic r/r RCCC suggest that more than one RCCC editing approach leads to cure, as few CRs were converted to long-term cCRs (two out of four CRs) as in angiosarcoma and cholangiocellular sarcoma, while two editing approaches were defined in mLCH (Reichle et al., 2007a). Heterogeneous long-term responses in r/r RCCC could be due to the absence of gp160 expression, a protein that mediates anti-tumor IFN responses, or RCCC subclones have a large genetic distance (Nanus et al., 1990; Sibai et al., 2022). In addition to the direct anti-tumor activity of interferon-I, it enhances CTL effector function via STAT3-activated granzyme B expression (Lu et al., 2019). Therefore, in the future, diagnosis of tumor tissue addiction-specific configuration dynamics of tumor-associated inflammation, likely including host responses, would provide a framework for pre-therapeutic optimization of tumor tissue editing schedules (Jong et al., 2024).

Pioglitazone/dexamethasone combination induces long-term cCR, CR, PR associated with long-term disease stabilization, by reprogramming tumor-associated inflammation and resolving or attenuating M-CRAC in multiple myeloma (MM), castration-resistant prostate cancer (CRPC) or r/r mLCH (Vogelhuber et al., 2015; Heudobler et al., 2019a; Harrer et al., 2023b). Thus, completely different histologies share, at least in part, clustering system vulnerabilities.

### Genome-agnostic classification of regulatory NOA circuitries of tumor tissue addictions

From a conceptual standpoint, NOA-based systems pharmacology is found on observations from randomized phase II and phase I/II trials (Table 4).

AML studies clearly demonstrate that significant clinical responses can be achieved using identical systems pharmacology approaches in genetically and molecularly highly heterogeneous AMLs (Heudobler et al., 2024a). Furthermore, genetic heterogeneity can be inferred from preceding mixed responses to targeted therapies. The consecutive achievement of continuous complete remissions with NOA-based systems therapy suggests that intratumoral genetic heterogeneity can be overcome, thereby again emphasizing the genome-agnostic approach.

We categorized NOA-based systems pharmacological approaches also as genome-agnostic if underlying tumor cell genetics were unknown but the histologies differed significantly, as in the case of r/r mLCH and r/r RCCC, but identical tumor tissue editing approaches induced cCR (Tabe 1; Figure 6).

One reason for the successful application of tumor tissue editing approaches is the genome agnostic activity of NOA based therapies in r/r metastatic neoplasias. The simultaneous targeting of NOAs in tumor and adjacent stromal cells facilitate major reprogramming of tumor-promoting hallmarks. Major tumor promoting hallmarks, inflammation, immunosurveillance, differentiation and tumor cell metabolism are constituted by cells of the tumor compartment, regardless of whether they are genetically altered or genetically normal but actively contributing to malignant behavior.

Therefore, editing therapies fulfill key therapeutic requirements to induce CR (in three tumor types) and cCR (in an additional five tumor types), even in seemingly incurable metastatic r/r neoplasms (Figure 6). The use of genome-independent tumor tissue editing approaches allows us to successfully tailor nuclear/cytokine receptor agonists to the specific addiction clusters (Reichle et al., 2007a; Harrer et al., 2022).

The addition of pioglitazone to metronomic chemotherapy in gastric cancer did not add any clinical benefit despite the involvement of PPARγ in the pathogenesis of gastric cancer (randomized phase II study) (Ezzeddini et al., 2021; Reichle et al., 2009). In high-grade gliomas only stabilization of disease was possible, while in hepatocellular carcinoma and NSCLC the best response was partial remission (PR) (Hau et al., 2007; Heudobler et al., 2021; Walter et al., 2017). As CR or cCR has been achieved in all other neoplasms, it is expected that the combination of receptor agonists in NSCLC, hepatocellular carcinoma and high-grade glioma can be optimized by adding pioglitazone and a third receptor agonist. In gastric cancer, novel receptor agonist combinations should be selected. Therefore, a classification of the respective regulatory NOA circuitries is not possible with the available outcome data (Table 5).

Transcriptionally regulated NOA circuitries can currently be classified according to the combination of receptor-triggered transcription factors targeted with receptor agonists, a systems pharmacological classification, or according to the stromal coregulated cancer hallmarks that are reprogrammed if CR or cCR can be achieved in principle, in a study cohort with metastatic r/r neoplasia (Luo et al., 2009) (Figures 2, 7). The possibility of a systems pharmacological tumor classification shows that pathways, cancer hallmarks and stress responses are similar in different tumor types, although each tumor can be classified as an individual tumor considering genetic alterations (Vogelstein et al., 2013).

### Uniform distant tumor response clusters

Only a few neoplasms can be cured in the metastatic stage with oncogene-addicted targets, e.g., in Philadelphia-positive chronic myeloid leukemia, ALK-positive neoplasms, neoplasms with high microsatellite instability (MSI-H) or deficient mismatch repair (dMMR) neoplasias due to excellent sensitivity to immune checkpoint inhibitors or CRs may be achieved with neurotrophic tyrosine kinase (NTRK) inhibitors in basket trials for pediatric and adult solid tumors with NTRK fusion (Thein et al., 2024; Yılmaz et al., 2024).

Genetic heterogeneity occurs primarily at metastatic sites, with additional transcriptional heterogeneity among neoplastic cells, organized into cancer cell states as a general characteristic of tumor tissue (Barkley et al., 2022; MacDonald et al., 2025).

The systematic control of metastatic r/r neoplasias without distinct oncogenic drivers by tumor tissue editing demonstrates that beyond cell-autonomous regulatory programs, classifiable large-scale communicatively linked transcriptional NOA circuitries, including the tumor stroma, are available at metastatic tumor sites and can be transcriptionally reprogrammed, importantly independent of genetic or transcriptional heterogeneity at metastatic sites. The evidence is CR and cCR across quite different neoplasias in the metastatic r/r stage in response to the concerted attack of the systems pharmacological approach, tumor tissue editing (Chen et al., 2024; Harrer et al., 2023b) (Table 5).

### Obstacles to the targeting of NOAs

Targeting individual NOA proteins faces three major therapeutic obstacles. (1) Individual NOA targets at metastatic sites prove to be heterogeneous like oncogenic aberrations and are therefore a major cause of treatment failure (Dagogo-Jack and Shaw, 2018). (2) Long-term tumor control is often limited, and (3) the induction of M-CRAC as a result of therapy-induced tumor stress responses is almost obligatory. Despite the successful use of NOA targets in metastatic r/r neoplasia, palliation remains the main therapeutic perspective (Chang et al., 2021; Hendriks et al., 2023).

Tumor tissue editing techniques take effective measures to treat metastatic r/r neoplasms by viewing NOAs as communicatively connected circuitries to establish new therapeutically relevant homeostatic equilibria.

A diagnostic problem is to uncover transcription factors that maintain hardwired NOA circuitries in cancer hallmarks and tumor stress responses for targeted reprogramming (Tao and Wu, 2023). Nuclear/cytokine receptors evade routine detection as NOAs and are distributed in all tumor cell compartments. Currently, only selective NOA proteins are studied in front-line diagnostics (Di Marco et al., 2024; Meyer et al., 2010; Hendriks et al., 2023).

Critical points for targeting NOA circuitries are the appropriate selection of combinations of nuclear/cytokine receptors and their selected on-topic activity in large-scale communicative circuitries of tumor tissue addictions. An experimentally less appreciated phenomenon is the co-regulatory activity profile of combined transcriptional modulation in the context of tumor systems, in particular, how the multifunctionality of NOA targets can be focused and edited, such as mTOR (Harrer et al., 2025).

Selection of the study population most likely to benefit from a particular tumor tissue editing approach is not possible, as NOA circuitries and the corresponding transcriptional profile have yet to be defined for individual patients (Meier-Menches et al., 2022; Heudobler et al., 2024a).

Since editing therapies are genome-agnostic, genetic heterogeneity in tumor cells plays a minor role in resistance mechanisms that may prevent the desired qualitative reprogramming of tumor tissues (Harrer et al., 2023b; Heudobler et al., 2024a) (Figure 1; Tables 1 and 4).

A critical obstacle for editing therapy could be a significant genomic distance between tumor subclones, which is associated with crucial differences in the activity of cancer traits due to the specialization of a tumor cell clone. This experimental finding cannot be excluded as a potential resistance mechanism relevant for clinical trials (Sibai et al., 2022; Harrer et al., 2023b). However, genomic distance between tumor subclones contributing to NOA circuitries that can no longer be targeted by a set of receptor agonists can be excluded as a common phenomenon in r/r HL and mLCH.

Since viability is a prerequisite for tumor growth, the transcriptomic landscape of each tumor should provide targetable transcriptional patterns accessible via receptor-induced nuclear/cytokine receptor NOAs.

### A major contribution to personalized oncology/hematology: editing tumor tissue

Following planned curative systemic therapy, the spectrum of potential toxicities that can significantly impact a patient’s quality of life and long-term health status can be enormous.

Advances in cancer therapies that take advantage of systems-based pharmacological approaches, such as tumor tissue editing techniques, can systematically minimize therapy-related toxicity combined with high specificity in targeting addicted tumor system circuitries (Harrer et al., 2023b; Harrer et al., 2023a) (Table 4).

Systems pharmacology approaches targeting tumor tissue addiction have “indirectly” led to long-term disease control or even CR and cCR via the “loop,” reprogramming of cancer hallmarks, deregulation of stress responses, and ultimately destabilization of the tumor tissue addiction context.

Therefore, editing approaches must not primarily address the question of who is eligible for therapy based on age or comorbidity. Toxicity can be mitigated simply by planned dose reductions that do not compromise response due to the biomodulatory activity of the editing drugs (Harrer et al., 2023b; Harrer et al., 2023a). The minimum activity of each drug in the editing schedules is not known in accordance with the scheduled dose reductions (Harrer et al., 2023b; Harrer et al., 2023a).

Normal tissues attenuate the induced transcriptional regulatory activity profile of nuclear/cytokine receptor agonists to clinically undetectable levels, as tumor-associated addictions are absent in normal tissues.

Scheduled dose reductions keep the toxicities of low dose metronomic chemotherapy low. Treosulfan, trofosfamide or capecitabine have shown very low emetogenic activity (Harrer et al., 2023b).

The modest toxicity patterns observed are known from the toxicity profiles of the individual drugs. The adverse event (AE) and serious adverse event (SAE) data monitored in the phase I/II studies do not indicate any novel toxicities associated with the drug combinations used. Tumor tissue editing approaches are associated with modest off-target effects (Harrer et al., 2023b; Harrer et al., 2023a).

Ubiquitously available receptor-induced transcription factors maintain NOA circuitries, as shown for those involved in tumor-associated inflammation. The inflammation-specific serum biomarker CRP enables CRP-guided precision medicine. Tumor response paralleled CRP response in RCCC, mLCH, HL and melanoma (Reichle et al., 2007a; Ugocsai et al., 2016; Harrer et al., 2022; Reichle et al., 2007b).

Addicted NOA circuitries are key targets that provide specific therapeutic access and are not available in normal tissues with organ-defined cell identity (Tables 3 and 5).

Tumor tissue editing approaches address the overarching goal of modern hematology and oncology to reduce off-target effects while improving treatment outcomes, here even with cCR in quite different histological r/r tumor stages (Tables 3 and 5). Tumor tissue editing approaches target transcriptionally accessible NOAs with analogs of natural receptor ligands at low doses compared to previously used high doses, e.g., of cytokines in RCCC (Atzpodien et al., 2006; Lüke et al., 2021; Harrer et al., 2023b).

Tumor tissue editing approaches represent a new therapeutic technology that enriches personalized medicine by altering tumor tissue addictions to three NOA levels integrated into cancer tissue addictions (Figure 3). Moreover, tumor tissue editing therapies are applicable on an outpatient basis, independent of comorbidities and sequence of pretreatments (Harrer et al., 2025). The cost of drugs used for editing is modest compared to available standard salvage therapies in metastatic r/r neoplasms.

## Discussion

The clinical studies presented address the question of whether targeting selected receptor-induced transcription factors as ubiquitously available NOAs by receptor agonists can therapeutically exploit the reprogramming of cancer hallmarks and stress responses in metastatic r/r neoplasia to achieve cure.

Combinations of receptor agonist-induced transcription factor NOAs revealed that the transcriptomic landscapes in tumor cell compartments operate tumor-specific tissue addictions and maintain therapeutically unlockable reprogramming profiles of cancer hallmarks exploitable as a therapeutic countermeasure to endogenously promoted oncogene-addicted transcriptional patterns that promote cancer hallmarks and stress responses: cross-tumor histology classifiable receptor-agonist combinations can genome-agnostically reprogram cancer hallmarks constituted with the support of the tumor stroma (Table 2).

The clinical trials summarized here reverse the current management tiers, now moving from bedside to bench, and are driven by the clinical motivation to successfully treat relapsed and refractory neoplasias with modest toxicity. The discussed mechanisms of action of tumor tissue editing therapies are clinically inferred or hypothesis-generating but have not yet been mechanistically determined.

The clinical “hardware” comprises the following: clinical outcome in thirteen histologically distinct r/r neoplasias, long-term tumor control, objective response, CR and cCR, modest response in high-grade gliomas and non-response to pioglitazone in gastric cancer.

Furthermore, if a “jump starter” targeting NOAs is introduced, such as low dose metronomic chemotherapy, azacitidine, or targeted therapies such as MEK inhibitors, pioglitazone can be successfully combined with additional transcriptional modulators to promote response in r/r neoplasias, again via NOA targets available across tumor tissues in r/r hematological neoplasias, cancers, and sarcomas. Reprogramming tumor promoting cancer hallmarks could be fixed as biological response surrogate.

Moreover, NOAs may be edited as rate-limiting targets (e.g., mTOR), as clinically indicated. Tumor-specific, clinically valuable clustering responses may be observed following tumor tissue editing, e.g., long-term maintenance of response after discontinuation of tumor tissue editing therapy, regain of hormone sensitivity and successful reuse of drugs that had previously failed with consecutive CR induction.

Thus, clinical data leads to novel models on how to use NOAs, as NOAs turn out as major targets to overcome MCRAC, passing the ball back to basic science to study the suggested modular organized NOA circuitries and to provide predictors of which transcriptional modulators can be combined most effectively, even in a genome-agnostic manner.

Tissue mass spectrometry imaging (MSI) would be an appropriate approach to studying the systems pharmacological basis of NOA circuitries (Bhatia et al., 2022; He Fuchu et al., 2024; Solier et al., 2023). Clinical trials involving MSI data in follow-up could clarify whether tumor tissue editing approaches could successfully adapt to existing NOA circuitries to provide a template for overcoming M-CRAC and controlling metastatic, recurrent or refractory neoplasias.

Comparing the heterogeneous accessibility of modularly organized NOA circuitries via tumor tissue editing approaches, including pioglitazone, with the oncogenic heterogeneity of the respective histological tumor types being treated provides insight into the suggested broad therapeutical accessibility of metastatic r/r neoplasias by tumor tissue editing approaches.

Considering the mutational load of the studied neoplasias, which is very high in NSCLC and melanoma (median 0.9 to 20 mutations per megabase), intermediate in gastric cancer, high-grade glioma, prostate cancer, hepatocellular carcinoma, cholangiocarcinoma and RCCC, in multiple myeloma and Hodgkin’s lymphoma and low (<0.9) in AML and mLCH, the achievement of an objective response, CR or cCR with the modular use of pioglitazone in tumor tissue editing approaches - with the exception for high-grade glioblastoma and no therapeutic impact of pioglitazone in gastric cancer -, suggests that the tool of modularly organized NOA-circuitries is heterogeneous but might be dramatically smaller and more structured than the highly heterogeneous tool of non-synonymous mutations when considering targets for resolving M-CRAC (Alexandrov et al., 2013; Vogelstein et al., 2013; Velasco et al., 2016; Marafioti et al., 1997; Rodriguez-Galindo and Allen, 2020).

Moreover, differentially operated tools promoting tumor-associated inflammation may share identical therapeutic accessibility of NOA circuitries via tumor tissue editing (Harrer et al., 2022; Reichle et al., 2007a). A recently experimentally uncovered, cross-tumor recurrent catalog of transcriptionally accessible gene modules for stress responses or cancer hallmarks might experimentally explain why a classification of NOA circuitries is possible (Barkley et al., 2022).

Tumor tissue editing reveals cross-tumor available receptor-driven transcription factors that maintain classifiable, hard-wired, networked NOAs that are integrated into cancer hallmarks and stress responses. Different histological tumor types share typical NOA circuitries, and identical histologies may express different types of networked NOAs (Harrer et al., 2022; Reichle et al., 2007a). However, unique NOA circuitries in histologically different tumor types do not imply that the expression of inflammatory features is identical at the molecular level (Reichle et al., 2007a; Harrer et al., 2022). Nevertheless, inflammation-associated NOA circuitries can be identified as classifiable and represent genome-agnostic transcriptionally accessible systems for systems pharmacological targeting.

In contrast to experimental findings that cancer cell states are highly plastic and cannot be defined as distinct cell types, CR and cCR responses to transcriptional editing suggest a unique transcriptional program that defines the reprogrammability of cancer hallmarks and stress responses via NOA circuitries also at metastatic sites (Barkley et al., 2022; Harrer et al., 2025).

Inactivation of a few cancer hallmarks is sufficient to achieve CRs and cCRs in the r/r stage of hematologic malignancies, cancers and sarcomas in the context of a therapeutically induced stress response and epigenetic modeling (Mrowka and Glodkowska-Mrowka, 2020; Harrer et al., 2023a).

Communicative reprogramming of tumor tissue vulnerabilities is best described by the term anakoinosis, as it describes the process, communicative reprogramming, mutual adaptation of reprogrammed cancer hallmarks, and deregulation of tumor stress responses, resulting in loss of tumor viability and, at best, cCR, even in high-risk patients (Hart et al., 2015; Pantziarka et al., 2019; Heudobler et al., 2019b; Heudobler et al., 2018b; Heudobler et al., 2024b). Anakoinosis is unique in that it works in a genome-independent manner by simply using tumor tissue processing capabilities to reprogram clustered NOA circuitries as cancer vulnerabilities (Figure 4).

Comparison of tumor tissue editing approaches now shows that receptor-triggered transcription factors are classifiable targets that induce anakoinosis. Cancer hallmarks and stress responses are always interdependent, which is why tumor tissue editing can successfully promote loss of tumor viability (Sibai et al., 2022).

Therapeutic transcriptional editing enables predictable perturbation of the transcriptomes of tumor disease hallmarks with minimal impact on normal tissues (Harrer et al., 2023b; Vervoort et al., 2022).

Anakoinosis directly counteracts the reprogramming capabilities of oncogenes by selectively editing communicatively cross-linked NOAs in tumor tissues while targeting commonly available receptor-driven transcription factors in the presence of stress instigation and epigenetic modeling (Harrer et al., 2023b).

The novel editing therapy completely changes our approach to the development of therapies for metastatic r/r neoplasms and underscores the efforts to successfully overcome the previously unresolved obstacles arising from therapy-induced M-CRAC. Histology, molecular genetics or oncogene dependency can be neglected to a certain extent as currently the most important stratification parameters for therapy selection in metastatic r/r neoplasia.

The new discriminatory parameter for the treatment of metastatic r/r neoplasia is the nature of addiction clusters, which are maintained as NOA circuitries by ubiquitous transcription factors (Table 3) (Siddiqui et al., 2018). As shown, tumor tissue addictions systematically provide transcriptional regulatory access to NOA circuitries in quite different tumor histologies, hematological neoplasms, sarcomas and cancers (Harrer et al., 2023b).

Thus, the maintenance of tumor viability is a comprehensive role of NOAs and NOA circuitries and an important hallmark of cancer (Sibai et al., 2022; Harrer et al., 2023b).

The main questions are how are NOA circuitries structured and to what extent are they therapeutically modifiable? What kind of transcriptional modulation to alter tumor phenotypes is pharmacologically possible? Is the rearrangement of agonists for receptor-triggered transcription factors sufficient to access addictive homeostatic equilibria to induce a cure, or do we need novel drugs with transcriptional access (Lüke et al., 2022)? In the future, the diagnosis and systematization of NOA circuitries involved in cancer characteristics and tumor stress responses could be as valuable for therapy selection as histological/molecular genetic tumor typing for the establishment of personalized hematology and oncology (Billingham et al., 2024; Nogales et al., 2022).

Approaching systemic tumor therapies from the other side, i.e., neglecting primarily the tumor genome, but therapeutically redirecting the functional status of generically available transcription factors with receptor agonists and redirecting oncogene-addicted transcription, would completely change the course of classical pharmacological research, which currently focuses on the target molecule, the animal model and clinical trials with accompanying multi-omics research.

Even if the editing approach itself does not trigger cCR, individual edited NOAs can emerge as target molecules that determine long-term outcome (Harrer et al., 2025). Thus, the possibilities to add a classical targeted therapy are manifold, also from a toxicity point of view, if one considers the toxicity of the entire treatment plan, e.g., in r/r HL (Lüke et al., 2021).

Solving the complexity of tumor treatment does not always mean going back to the oncogenic roots, but recognizing that tumor tissue must mobilize the vast number of wild-type genes to generate NOAs, NOA circuitries and tumor tissue addictions, a tool that provides genome-agnostic pharmacological targets for tumor tissue editing techniques (Swanton et al., 2024; Heudobler et al., 2024b; Harrer et al., 2023b; Nogales et al., 2022).

NOA circuitries dealing with cancer hallmarks and stress responses can be transcriptionally separated from oncogene-driven circuitries by systems pharmacology approaches. Tumor tissue editing thus fundamentally alters cancer architecture, apparently independently of the spatial landscape of cancer hallmarks and tumor stress responses (Sibai et al., 2022).

Therapeutic outcomes in metastatic r/r neoplasia with tumor tissue editing go beyond palliation compared to the established therapeutic approaches using inhibitors of tissue-specific master transcription factors, androgen, estrogen and progesterone receptors in recurrent hormone-dependent cancers (Heudobler et al., 2024b; Kim et al., 2025). However, both approaches can be complemented by NOA-targeted therapies. Following tumor tissue editing, single edited NOAs emerge as key targets for achieving cCR (Bono et al., 2020; Lloyd et al., 2024; Lüke et al., 2021).

Systems pharmacological therapy techniques, such as tumor tissue editing, are instrumental in advancing the field of treatment approaches for the long-term control and cure of metastatic r/r neoplasias.
